# International oil price uncertainty and enterprise investment efficiency: An empirical research of listed companies in China

**DOI:** 10.1371/journal.pone.0299084

**Published:** 2024-10-10

**Authors:** Yonghui Zhan

**Affiliations:** Gas Company of Sinopec, Beijing, China; University of Almeria: Universidad de Almeria, SPAIN

## Abstract

This article investigates the effect of international oil price uncertainty on investment efficiency using quarterly data of Chinese A-share listed companies from 2011 to 2020. It is discovered that uncertainty about oil prices increases principal-agent conflicts and information asymmetry in enterprises, leads to more inefficient investment, and ultimately lowers firms’ investment efficiency. Furthermore, China’s unique property rights foundation, firm size, and life cycle limit the aforementioned consequences. Additional investigation reveals that by raising companies’ cash reserves, oil price uncertainty exacerbates overinvestment behavior. The study presented in this article can serve as a guide for businesses looking to make the best decisions in the context of volatile global oil prices and support their long-term, stable development.

## 1. Introduction

Due to its characteristics as a commodity, crude oil, as a major source of energy, has the ability to convey the risk of fluctuating oil prices to different industries through the production mechanism, which has an impact on corporate investment [1], financial performance [2], cash reserves [3], capital structure [4], driving up foreign exchange expenditure growth, creating inflationary pressures, and potentially wreaking havoc on the economy [5]. Additionally, the global oil price supply and demand tensions have resurfaced, the international oil price is uncertain or has entered a new phase due to the development of new energy technology, the geopolitical deadlock, the sudden emergence of a new epidemic, and other factors [6, 7]. At the same time, China’s economy is more vulnerable to the negative effects of changes in oil prices due to its low crude oil energy utilization rate, little improvement in the rate of new energy substitution, high external dependency on crude oil, and low crude oil energy utilization rate [8]. One of the key ways that the macroeconomy is impacted by crude oil prices is through enterprise investment. Oil price fluctuations create significant uncertainty regarding future costs, demand, profitability, and other factors, making it challenging for businesses to decide how much money to invest [9], which reduces the effectiveness of their investments. As a result, a reasonable response to the impact of oil price uncertainty risk on investment efficiency can help improve enterprise efficiency and promote the healthy development of the economy.

How to maximize corporate investment efficiency has grown to be a crucial component of fostering corporate transformation and upgrading in the current environment of China’s efforts to promote high-quality growth. In addition to internal characteristics like corporate governance, management, and information disclosure [10, 11], the factors that limit and improve enterprise investment efficiency also include the mechanism of an uncertain external environment [12, 13], and scholars have already conducted preliminary investigations into uncertainty. Li Mei and Takayama Hsing (2014) [14] discovered that market uncertainty has the opposite effect, tending to retain capital in a competitive market, leading to underinvestment, whereas technological uncertainty encourages firms’ product reforms and enhances the efficiency of firms’ investment in innovation. According to Bradley et al. (2016) [15], firms are motivated to improve investment efficiency during times of high policy uncertainty because investors demand high returns to make up for the high risk brought on by policy uncertainty. Yeh (2017) [16] notes that management turns to private information more when conditions are uncertain, and that this "pandering" drive causes businesses to overinvest, which lowers investment efficiency. Hou et al. (2021) [17] found that economic uncertainty increases management’s defensive mentality and decreases investment efficiency using data from a sample of listed energy and power companies in China. Overall, these studies accurately measure the different uncertainties that the company faces and empirically test how those uncertainties affect the company’s investment efficiency. However, despite being a significant branch of uncertainty, oil price uncertainty has not yet received much attention from academics in their study of the company’s investment efficiency. To better understand how the external environment affects micro-enterprises, this article attempts to analyze the relationship between corporate investment efficiency and the mechanism of oil price uncertainty.

This study contributes to a deeper knowledge of how oil price uncertainty influences company development by elucidating the impact of oil price uncertainty on the economy by moving the focus from investment activity to investment efficiency, based on the findings of prior studies. Understanding the relationship between oil price uncertainty, overinvestment, and cash holdings is strengthened by investigating the distinct effects of oil price uncertainty on investment efficiency and identifying the mediating transmission mechanism of cash holdings on overinvestment in oil price uncertainty. The literature review and research hypotheses are covered in the second section of the article; the research design is covered in the third; the results of the empirical study are reported and analyzed in the fourth; additional analyses are covered in the fifth; and the publication’s conclusion is covered in the sixth.

## 2. Literature review and hypotheses development

### 2.1 Factors affecting investment efficiency

Numerous factors influence investment, but at this point the level of internal company characteristics is the key concern. First, managers, who serve as the general helmsmen of business operations, have a variable impact on the effectiveness of corporate investment due to their overconfidence [18] and individual differences in competence [19], tenure [20], gender, age, and educational background [10]. Second, effective agency problem mitigation and weakening of the firms’ inefficient investment behavior can be achieved through high-quality internal control [21], effective disclosure of social responsibility information [11], and high level surplus management [22]. In addition, dividend payments have an impact on investment efficiency through enterprises’ internal cash flows when combined with financing restrictions [23]. At the same time, some scholars note that the business activities of enterprises are not independent of the external environment, and point out that the external environment has a significant impact on investment efficiency, and that this impact is the reason that research has turned its attention to studying the external environment and investment efficiency. In the study of uncertainty and investment efficiency-related research, Shen Huihui (2012) [12], Xu Qian (2014) [24], and other researchers noted that environmental uncertainty enhancement, on the one hand, exacerbates the degree of information asymmetry of the enterprise, leading to overinvestment; on the other hand, increases the possibility of risk avoidance of the management of the enterprise, promoting underinvestment behavior. Xie Weifeng and Chen Shenghong (2020) [25] found that rising economic policy uncertainty in China leads to a decline in trust between the principal-agent parties, a moral crisis, and the reverse behavior of the management, which leads to investment inefficiency. According to Gu Haifeng and Zhu Huiping (2021) [9], the higher the level of uncertainty, the harder it is for management to accurately understand market conditions. As a result, management will reduce the size of existing investments by avoiding risks out of prudential considerations, which in turn hinders the firms’ ability to invest effectively. Wu et al. (2021) [1] and Cao et al. (2020) [26] observe that oil price uncertainty alters businesses’ investment efficiency by impacting the prospective capital return and altering investment sensitivity.

### 2.2 Economic consequences of oil price uncertainty

The economic consequences of international oil price uncertainty are mostly grounded in micro firms and have been richly explored. Increased oil price uncertainty decreases firm performance [2], increases cash holdings [3], decreases firm leverage [4], and increases firms’ cost stickiness [27], impact of price on stock market [28], country’s economic performance [29, 30],but there are two main theories regarding the relationship between investment and oil price uncertainty. The first viewpoint is based on real options theory, which contends that when a firm’s investment is irreversible (or at least partially irreversible), the rise in oil price uncertainty increases the value of the option to delay the investment, and the greater the opportunity’s ability to produce expected cash flows, the greater the likelihood that the firm will choose to postpone the investment [31–34]. Based on the theory of strategic growth options and compound options, another perspective contends that there is a U-shaped correlation between corporate investment and oil price uncertainty, and that once a certain point in time is reached, further increases in uncertainty force an increase in investment [35, 36].

In conclusion, most research on the factors influencing investment efficiency is still conducted from the internal perspective of enterprises, such as management, internal control, information disclosure, and the perspective of economic policy uncertainty, and only a small number of studies examine the effects of enterprise production factor market uncertainty on investment efficiency. For the economic consequences of international oil price uncertainty, the research results of oil price uncertainty and enterprise investment are fruitful, but they are limited to the level of the impact of the volume of investment and have not been refined to the level of investment efficiency. Oil is not only a strategic resource for firms, but also an important economic variable in the factor market. The volatility of oil will unavoidably influence management’s decision-making tendencies and the formulation of corporate strategies, which further influences the optimal investment of organizations [37]. Therefore, it is of great theoretical and practical significance to study the problems of investment efficiency and oil price uncertainty.

### 2.3 Hypotheses development

The lack of key information, the lack of resources and the absence of external regulation triggered by oil price uncertainty leads to an increase in the degree of information asymmetry and agency problems among firms, and investment and financing decisions are more likely to deviate from the optimal level. Therefore, oil price uncertainty has the following two levels of impact on investment efficiency:

On the one hand, the impact based on the underinvestment perspective. Firstly, from the operational perspective, the increase in oil price uncertainty raises the option value of waiting for investment, and due to the irreversibility of corporate investment, if the investment decision is made at this time, the firm’s expected return on assets cannot reach the sum of the actual cost of the investment and the option value of waiting for the disappearance of uncertainty [1, 26]. Therefore, enterprises will weaken the scale of investment to mitigate the impact of oil price uncertainty on themselves, which will result in the phenomenon of underinvestment and worse overall investment efficiency. Second, from the perspective of capital, the uncertainty surrounding oil prices reduces market demand, which lowers enterprise endogenous capital [32, 33] and strict restrictions on bank credit supply make it difficult for businesses to obtain external loans [4, 38], which forces businesses to experience underinvestment and reduce investment efficiency. Third, from the perspective of the management, the risk borne by the management to invest in the period of oil price uncertainty is not matched with the benefits obtained from successful investment, and the management has insufficient motivation to improve investment efficiency. Oil price uncertainty weakens the link between management compensation and business performance, and does not motivate management to put in extra effort through business performance. Uncertainty limits investment possibilities and, thus, management discretionary power [39], so if management decides to make investments now, it will incur higher private costs and risk pressures in the event that the investments fail. The uncertainty of oil prices leads to the distortion of financial information obtained by enterprises [25]. Using this confusing information, the risk of management’s investment decision failing is now rising, the level of trust between the principal-agent parties is declining, and management will fully bear the risk of an unsuccessful investment. Therefore, based on the consideration of maximizing its interests, the manager is more willing to reduce the scale of corporate investment in the face of the risk of oil price uncertainty, and the enterprise shows the tendency towards underinvestment. Combined with the above research findings, enterprises are more inclined to reduce their willingness to invest in the face of oil price uncertainty, which inhibits their investment efficiency.

On the other hand, the impact based on the overinvestment perspective. First, it is challenging to disclose the behavior of business management during the period of oil price uncertainty due to the absence of external stakeholder oversight [25]. According to the information asymmetry theory, the firm’s management has access to adequate information about the transaction and can profit from it compared to the firm’s external information consumers. As a result, during a time of fluctuating oil prices, management uses the information in its possession to deviate from management objectives, harming shareholders’ rights and interests, and carrying out overinvestment motives. Second, oil price uncertainty gives management an excuse to rationalize investment failures. Management can attribute investment failure to the risks associated with oil price uncertainty and reduce the responsibility it needs to bear [16]. Therefore, management may create a business empire during periods of uncertain oil prices, which might result in the phenomenon of excessive corporate investment. Third, it is unlikely that top management will be fixed in one position for a long period, and the economic consequences of investment projects have a lag. The absence of external regulators and principal-agent conflicts caused by oil price uncertainty makes it easier for management to manipulate accounting information, optimize the content of statements, pursue short-term profits, and enhance their reputation and wealth, bringing about the problem of overinvestment. In summary, this paper proposes the following hypotheses:

H1: Oil price uncertainty exacerbates firms’ inefficient investment behavior and inhibits their investment efficiency.

Capital is the material basis for enterprises to make investments. To counteract the risks associated with oil price uncertainty, enterprises tend to change their cash holdings, while their endogenous capital in turn directly affects investment decisions and alters investment efficiency. First, from the perspective of underinvestment, because of the drop in consumer demand brought on by oil price uncertainty, the rise in corporate spending to manage oil price risk, and the decline in external credit organizations’ willingness to provide funds, enterprises typically increase their cash reserves during the time of oil price uncertainty, which lowers the propensity to invest in businesses and exacerbates the problem of underinvestment [2]. In addition, based on the information asymmetry theory, to avoid the occurrence of adverse selection, which is detrimental to the interests of lending organizations, the strength of corporate financing constraints increases, and capital becomes a scarce resource for enterprises. As a result, management prefers to invest in R&D and innovation projects with higher benefits, crowding out the amount of investment in corporate entities [9], leading to underinvestment in enterprises. From the perspective of overinvestment, businesses set aside a lot of internal cash as a precaution to deal with the danger of fluctuating oil prices, but the more internal cash maintained by businesses, the more opportunities there are to increase the firm’s worth through investment [40, 41]. According to the free cash flow hypothesis, the funds left over after taking care of operational and development costs for the business and making the appropriate investments are at the manager’s discretion. As a result, during the period of fluctuating oil prices, the management can invest the discretionary excess cash holdings in specific high-risk or unrelated projects to increase the value of the company and generate excess returns to meet their own consumption needs. Furthermore, out of consideration of their prestige and reputation, management makes investment decisions that favor their strengths and overinvest in the short term to improve the firm’s performance in order to increase their bargaining power with shareholders during the period of uncertain oil prices. Accordingly, this paper proposes the following hypotheses:

H2a: Oil price uncertainty exacerbates underinvestment behavior, partly through the mediating effect of increased cash holdings.
H2b: Oil price uncertainty exacerbates overinvestment behavior, partly through the mediating effect of increased cash holdings.

**Fig 1:**
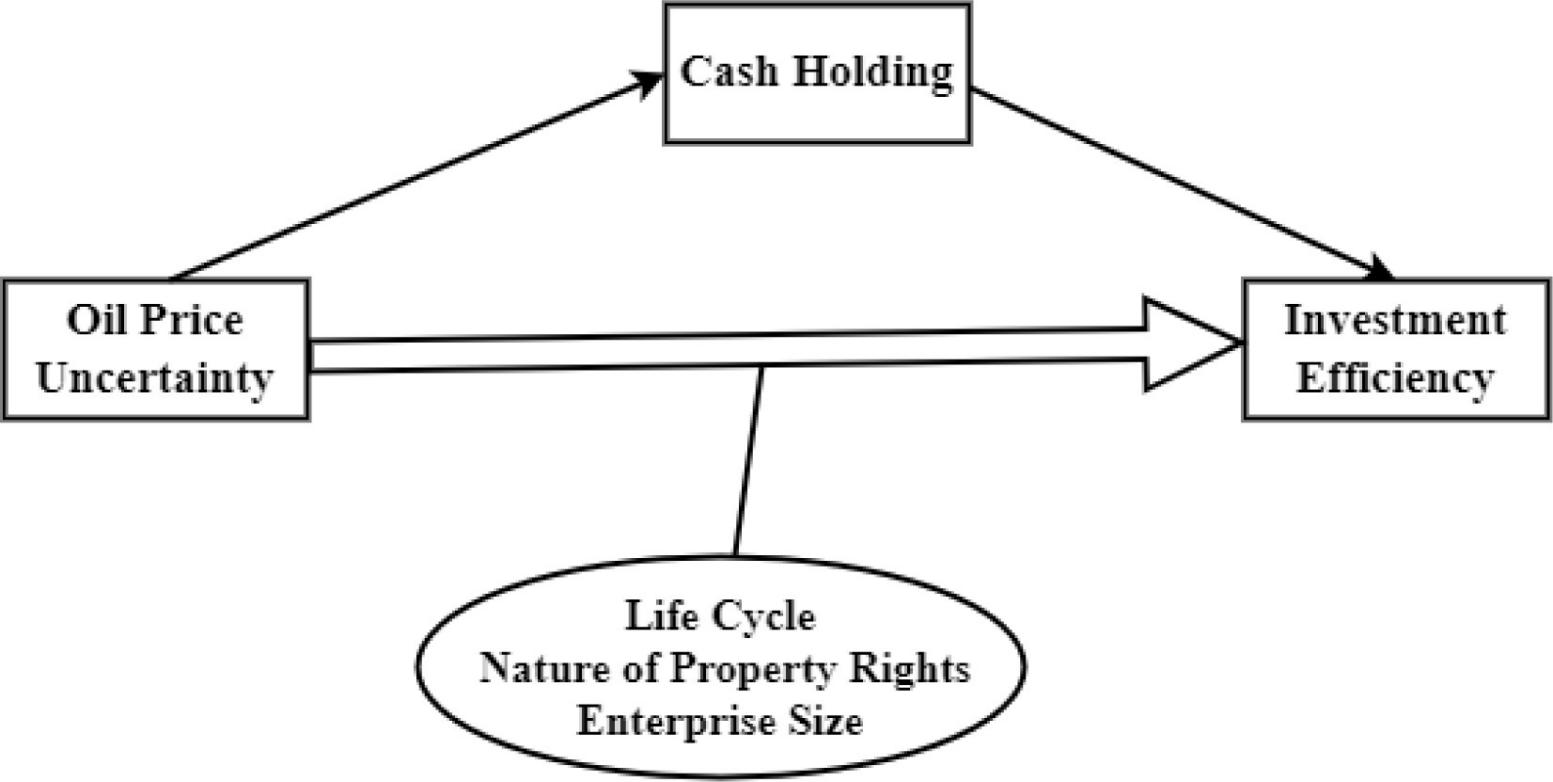
Framework for the study of oil price uncertainty and firms’ investment efficiency.

Based on the above research analyses, the research in this paper is shown in Fig 1.

## 3. Methods

### 3.1 Data and sample

This study uses Chinese A-share-listed companies from Q1 2011 to Q4 2020 as its research sample, which spans a number of oil price shock episodes. The U.S. Energy Administration (EIA) provided the WTI daily light crude oil spot price data used in the analysis, and the quarterly financial data of the firms is from the database CSMAR. In addition, to effectively eliminate the interference of other irrelevant factors, the original sample is processed according to the following principles: (1) exclude listed companies containing the words "ST, *ST"; (2) exclude the data of companies in the financial, education, health, service, and general industries, which have a relatively small relationship with the volatility of oil prices; (3) exclude the data of companies with zero or negative total assets; (4) exclude the data of companies listed for shorter than 1 year; (5) to reduce the impact of outliers on the analysis results, all continuous variables in the sample are treated with upper and lower 1% Winscribe. Finally, 91016 observations are obtained. The data processing and result output of this paper are executed by Stata17.0 software.

### 3.2 Variables

#### (1) Global oil price uncertainty

Two methods are more commonly used in the existing literature to measure oil prices uncertainty: the conditional standard deviation that results from forecasting changes in daily returns using a GARCH model [1, 32] and the standard deviation of the daily return on crude oil prices [33]. In comparison, the GARCH model is better able to explain the phenomenon of clustering of changes in crude oil returns, so this paper adopts the GARCH (1, 1) model as the explanatory variable of the regression.

#### (2) Investment efficiency

Given the relevant studies conducted by other scholars [9, 16, 42], this study intends to use the absolute value of the residuals calculated by the Richardson regression model as a measure of the enterprise’s investment efficiency. This indicator is inverted, meaning that the higher the value, the greater the degree of divergence from the enterprise’s optimal investment, and the lower the enterprise’s investment efficiency. The Richardson model is shown in (1):

$${Inv}_{i,t}=\beta_{0}+\beta_{1}{Inv}_{i,t-1}+\beta_{2}Q_{i,t-1}+\beta_{3}{Cash}_{i,t-1}+\beta_{4}{Lev}_{i,t-1}+\beta_{5}{Return}_{i,t-1}+\beta_{6}{Size}_{i,t-1}+\beta_{7}{Age}_{i,t-1}+\sum Ind+\sum Quartetly+\varepsilon$$ (1)

In model (1), *i* denotes the *i*th firm; *t* denotes the year; *Inv*_*it*_ represents the investment expenditures of company *i* in year *t*, measured by the ratio of cash paid for the purchase and construction of fixed assets, intangible assets and other long-term assets to total assets at the beginning of the period; *Q* denotes growth, measured by Tobin’s *Q*; Lev denotes capital structure, measured by the ratio of liabilities to assets; and Cash measures the level of cash holdings by the ratio of money funds to total assets at the beginning of the period; Return denotes the annual rate of return on the stock of the firm in which the cash dividends are reinvested; Size measures the size of the firm in terms of the natural logarithm of the firm’s total assets; and Age denotes age, measured in terms of the natural logarithm of the firm’s years on the market.

### 3.3 Model specification

To test the research hypothesis H1 as described in the previous part, the research model (2) is constructed. *Inveff* represents the efficiency of corporate investment, *GARCHOIL* represents the uncertainty of the international oil price, and the Industry and Time dummy variables are introduced to represent the industry, quarter, and year fixed effects of the model, respectively.


$${Inveff}_{i,t}=\alpha +\beta_{1}{GARCHOIL}_{i,t-1}+\gamma {Controls}_{i,t-1}+\sum Ind+\sum Time+\varepsilon_{it}$$ (2)

To further verify the mediating effect of cash holdings in oil price uncertainty and corporate inefficient investment, the step-by-step method is used to establish the following recursive model. In the first step, model (3) is established to test whether the regression coefficient of oil price uncertainty and inefficient investment is significant; in the second step, model (4) is established to verify whether the regression coefficient of oil price uncertainty on cash holdings is significant; and in the third step, the significance of the regression coefficients of oil price uncertainty and cash holdings, respectively, on inefficient investment is tested, as shown in model (5). Where *Inveff* in models (3)-(5) includes underinvestment (*Underinveff*) as well as overinvestment (*Overinveff*), and other control variables are described in Table 1.


$${Inveff}_{i,t}=\alpha +\beta_{1}{GARCHOIL}_{i,t-1}+\gamma {Controls}_{i,t-1}+\sum Ind+\sum Time+\varepsilon_{it}$$ (3)

$${Cash}_{i,t}=\alpha +\beta_{1}{GARCHOIL}_{i,t-1}+\gamma {Controls}_{i,t-1}+\sum Ind+\sum Time+\varepsilon_{it}$$ (4)

$${Inveff}_{i,t}=\alpha +\beta_{1}{GARCHOIL}_{i,t-1}++\beta_{2}{Cash}_{i,t}+\gamma {Controls}_{i,t-1}+\sum Ind+\sum Time+\varepsilon_{it}$$ (5)

**Table 1:** Definition and description of variables.

Variable category	Variable symbol	Variable description
implicit variable	*Inveff*	Absolute value of model (1) regression residuals
	*Overinveff*	Model (1) regression residuals for positive observations
	*Underinveff*	Model (1) regression residuals are negative in absolute terms
independent variable	*GARCHOIL*	Results of GARCH (1,1) calculations
intermediary variable	*Cash*	Cash at end of quarter in cash equivalents/total assets at beginning of period
control variable	*ROA*	Quarter-end net profit/total assets
	*Lev*	Quarter-end total liabilities /total assets
	*Colla*	Quarter-end net fixed assets /total assets
	*Adm*	Quarter-end administrative expenses/operating income
	*Size*	Natural logarithm of quarter-end total assets
	*Top*1	Number of shares held by the largest shareholder /total number of shares of the enterprise
	*Id*	Ratio of the number of independent directors to the number of board of directors
	*Dboard*	Number of Board of Directors
	*Sboard*	Number of supervisory boards
	*Dual*	If the chairman of the board and the general manager are both appointed, the value is 1, and vice versa is 0.
	*Age*	The natural logarithm of the difference between the current year and the year of listing

## 4. Empirical results

### 4.1 Descriptive statistics

Table 2 shows the descriptive statistical values of the variables related to the empirical test. Among 91,016 observations, the proportion of underinvestment samples is as high as 63%, which indicates that the underinvestment behavior of most enterprises is more serious and that there is still room for improvement in enterprise investment efficiency. The investment efficiency (*Inveff*) ranges from 0.0001 to 0.056, which means that there are large differences in the investment efficiency of different enterprises in different periods, and the standard deviation of 0.0098 is the same as the mean value of 0.0086, which indicates that the inefficient investment behavior of listed companies is common. The maximum value of oil price uncertainty (*GARCHOIL*) of 0.0086 is eight times more than its minimum value, which indicates that the quarterly oil price fluctuation between the samples is large, and further confirms the practical significance of exploring the uncertainty of oil price on investment efficiency in different periods.

**Table 2:** Descriptive statistics.

Variable	Mean	Std.dev	Min	Median	Max
*Inveff*	0.0086	0.0098	0.0001	0.0055	0.0560
*Overinveff*	0.0118	0.0144	0.0001	0.0064	0.0725
*Underinveff*	0.0068	0.0060	0.0001	0.0053	0.0335
*GARCHOIL*	0.0009	0.0017	0.0001	0.0005	0.0086
*SDOIL*	0.0251	0.0205	0.0086	0.0196	0.0948
*Cash*	0.0471	0.1118	-0.1759	0.0153	0.5584
*Lev*	0.4184	0.2086	0.0446	0.4096	0.8817
*Q*	2.0171	1.2258	0.8742	1.6189	7.9079
*CF*	0.0110	0.0375	-0.1030	0.0103	0.1341
*ROA*	0.0111	0.0159	-0.0448	0.0092	0.0674
*Colla*	0.2110	0.1582	0.0021	0.1784	0.6911
*Adm*	0.0953	0.0807	0.0083	0.0747	0.5068
*Size*	22.1922	1.2929	19.8607	22.0062	26.1439
*Age*	1.9835	0.9401	0.0000	2.1972	3.2581
*Top*1	35.0633	14.9011	8.7869	33.2235	74.9648
*GDP*	0.0263	0.1193	-0.2568	0.0642	0.2103
*Id*	0.3748	0.0532	0.3333	0.3333	0.5714
*Dboard*	8.6054	1.6881	5	9	15
*Sboard*	3.5562	1.0195	3	3	7
*Dual*	0.2682	0.4430	0	0	1
*SOE*	0.3733	0.4837	0	0	1

### 4.2 Results of regression analysis

According to column (1) of Table 3, oil price uncertainty (*GARCHOIL*) has a significant incentive effect on investment efficiency (*Inveff*). Since investment efficiency (*Inveff*) is an inverse indicator, this positive correlation can be seen in the fact that, as international oil price uncertainty increases, the value of *Inveff* decreases, which means that the oil price uncertainty inhibits the enterprise’s ability to invest effectively. This finding supports the paper’s main hypothesis, H1, which was also confirmed.

**Table 3:** Oil price uncertainty and investment efficiency regression test results.

Variable	(1)	(2)	(3)	(4)	(5)	(6)	(7)
	*Inveff*	*Underinveff*	*Cash*	*Underinveff*	*Overinveff*	*Cash*	*Overinveff*
*GARCHOIL*	0.109***	0.091***	2.333***	0.092***	0.158**	2.333***	0.138**
	(4.19)	(4.81)	(10.12)	(4.90)	(2.45)	(10.12)	(2.14)
*Cash*				-0.001**			0.007***
				(-2.55)			(5.43)
*Lev*	0.003***	0.002***	-0.022***	0.002***	0.004***	-0.022***	0.004***
	(12.59)	(12.83)	(-11.29)	(12.62)	(7.54)	(-11.29)	(7.69)
*Q*	0.000***	0.000***	0.006***	0.000***	0.001***	0.006***	0.001***
	(14.03)	(14.99)	(14.82)	(15.13)	(10.53)	(14.82)	(10.11)
*CF*	0.001	0.006***	0.010	0.006***	-0.010***	0.010	-0.010***
	(1.08)	(8.35)	(1.06)	(8.37)	(-4.04)	(1.06)	(-4.05)
*ROA*	0.022***	0.007***	0.361***	0.008***	0.014**	0.361***	0.012**
	(9.11)	(3.71)	(14.90)	(3.88)	(2.33)	(14.90)	(1.97)
*Colla*	0.006***	0.002***	-0.028***	0.002***	0.007***	-0.028***	0.007***
	(23.58)	(12.00)	(-14.06)	(11.86)	(11.31)	(-14.06)	(11.59)
*Adm*	0.000	0.001**	0.003	0.001**	-0.001	0.003	-0.001
	(0.01)	(2.30)	(0.61)	(2.30)	(-0.93)	(0.61)	(-0.97)
*Size*	-0.000***	-0.000**	-0.002***	-0.000**	-0.001***	-0.002***	-0.001***
	(-5.81)	(-1.98)	(-5.94)	(-2.02)	(-7.70)	(-5.94)	(-7.49)
*Age*	-0.002***	-0.002***	-0.000	-0.002***	-0.002***	-0.000	-0.002***
	(-41.83)	(-47.54)	(-0.06)	(-47.55)	(-19.49)	(-0.06)	(-19.64)
*TOP*1	-0.000	-0.000	0.000***	-0.000	0.000	0.000***	0.000
	(-1.07)	(-0.65)	(7.73)	(-0.58)	(1.34)	(7.73)	(1.09)
*Id*	0.002**	0.000	0.013**	0.000	0.005***	0.013**	0.005***
	(2.45)	(0.50)	(2.23)	(0.52)	(2.84)	(2.23)	(2.78)
*Dual*	0.001***	0.000***	-0.001	0.000***	0.001***	-0.001	0.001***
	(6.76)	(5.00)	(-1.62)	(4.99)	(3.10)	(-1.62)	(3.17)
*Dboard*	-0.000	-0.000**	0.001***	-0.000**	-0.000	0.001***	-0.000
	(-0.99)	(-2.12)	(5.52)	(-2.08)	(-0.02)	(5.52)	(-0.23)
*Sboard*	-0.000***	-0.000	0.001***	-0.000	-0.000	0.001***	-0.000
	(-2.67)	(-0.37)	(3.31)	(-0.35)	(-0.81)	(3.31)	(-0.95)
*GDP*	-0.002	-0.001	-0.095***	-0.001*	-0.003	-0.095***	-0.002
	(-1.42)	(-1.58)	(-10.18)	(-1.68)	(-0.84)	(-10.18)	(-0.58)
_*cons*	0.016***	0.012***	0.170***	0.012***	0.026***	0.170***	0.025***
	(20.02)	(20.73)	(23.58)	(20.89)	(12.62)	(23.58)	(12.06)
*Industry*/*Time*	Yes	Yes	Yes	Yes	Yes	Yes	Yes
N	91016	57719	91015	57718	33297	91015	33297
F	234.178	233.682	823.140	227.988	100.545	823.140	98.823
R^2^	0.0977	0.1541	0.3635	0.1542	0.0955	0.3635	0.0974

Note: ***, **, and * denote significance at the 1%, 5%, and 10% level, respectively (the same below).

Hypothesis H1 is tested separately in the Overinvestment (*Overinveff*) as well as Underinvestment (*Underinveff*) subsamples and the results are shown in columns (2) and (5) of Table 3. Although the significance of the coefficients of the two groups is different, they both meet the requirement of statistical significance. The negative effect of oil price volatility affecting investment efficiency holds for both sub-samples, and this negative effect is more pronounced in the underinvestment sample. Overall, the regression results in Table 3 agree with the paper’s hypothesis H1, which states that oil prices uncertainty exacerbates overinvestment and underinvestment and reduces firms’ ability to invest efficiently.

Columns (2)-(7) of Table 3 verify the mediating effect of cash holdings between oil price uncertainty and investment efficiency, with the first three columns showing the regression results of oil price uncertainty on underinvestment through cash holdings. Column (2) shows that the coefficient of oil price uncertainty and underinvestment is significantly positive at the 1% level, which states that oil prices uncertainty exacerbates underinvestment significantly. Column (3) shows that for every one-unit increase in oil price uncertainty, cash holdings also increase by 2.333 units. However, the coefficient of column (4) indicates that cash holdings make firms underinvest weaker, and the sign of the direct effect of oil price uncertainty and underinvestment (0.092) is opposite to the sign of the indirect effect of cash holdings (2.333×(-0.001)), which means that cash holdings have a "masking effect" on oil price uncertainty and underinvestment, and the hypothesis H2a is not valid.

The last three columns of Table 3 show the effect of oil price uncertainty on overinvestment through cash holdings. Column (5) shows that the negative effect of oil price volatility on overinvestment holds at the 1 percent level, which states that oil prices uncertainty has a significant inhibitory effect on underinvestment. Column (7) shows that the coefficients of cash holdings and overinvestment are significantly positive at the 5 percent level, and the coefficients of oil price uncertainty and overinvestment are significantly positive at the 5 percent level. So, hypothesis H2b holds true. For precautionary reasons, higher oil price uncertainty makes firms choose to hold more cash reserves, and a large amount of cash reserves provides firms with the possibility of overinvestment. In other words, the transmission path of "oil price uncertainty—cash holding—overinvestment—investment efficiency" is reasonable.

### 4.3 Robustness tests

#### (1) Replacing the oil price uncertainty variable

Drawing on the research design of Phan (2019) [33] on oil price uncertainty, this paper re-measures quarterly oil price uncertainty using the standard deviation of the daily rate of return on the oil price, which is calculated in Eq (5). Where *N* represents the actual number of trading days in quarter t and *R*_*i*_ is the daily return on the ith working day of that quarter. The quarterly oil price uncertainty for quarter t is:

$${SDOIL}_{t}=\sqrt{\frac{1}{N-1}\sum_{i=1}^{N}{{(R}_{i}-E(R))}^{2}}$$ (6)

where $E(R)=\sum_{i=1}^{N}R_{i}/N$ is the arithmetic mean of daily returns during the quarter. The robustness test results are shown in the first column of Table 4, which essentially support the original regression.

**Table 4:** Robustness tests.

Variable	*Inveff*1	*Inveff*2	*Inveff*2	*Inveff*3	*Inveff*3	*Inveff*4	*Inveff*4
*GARCHOIL*		0.083***		0.116***		0.063*	
		(2.69)		(3.47)		(1.72)	
*SDOIL*	0.009***		0.007***		0.009***		0.006**
	(4.40)		(3.08)		(3.57)		(2.05)
_*cons*	0.016***	0.020***	0.020***	0.019***	0.018***	0.022***	0.022***
	(19.57)	(20.91)	(20.61)	(20.05)	(19.66)	(20.92)	(20.79)
*Controls*	Yes	Yes	Yes	Yes	Yes	Yes	Yes
*Industry*/*Time*	Yes	Yes	Yes	Yes	Yes	Yes	Yes

### (2) Replacing the investment efficiency variable

Referring to the study of Biddle (2009) [43], the absolute value of the residuals from the regression of model (6) is used to measure the investment efficiency of the firm (*Inveff*2):

$${Inv}_{i,t}=\beta_{0}+\beta_{1}{Growth}_{i,t-1}+\varepsilon$$ (7)

Meanwhile, considering the possible impact of measurement bias, the value of cash paid for the purchase and construction of fixed assets, intangible assets and other long-term assets minus the net cash recovered from the disposal of fixed assets, intangible assets and other long-term assets is used to divide the total assets at the beginning of the period [9] to replace the *Inv*_*it*_ in the models (1) and (6). It means we obtain new variables for measuring investment efficiency (*Inveff*3,*Inveff*4).

According to the results in columns 2–4 of Table 4, the regression results obtained for *Inveff*2,*Inveff*3, and *Inveff*4 are generally consistent with the previous results. So, the conclusions of this paper can be considered robust.

## (3) Replacing the regression models

To mitigate and eliminate the endogeneity problem caused by omission, in addition to the baseline regression lagging the explanatory variables by one period, this paper further selects the Tobit model to retest the above regressions. The results are shown in Table 5 and the results of the previous regression are consistent.

**Table 5:** Tobit model tests.

Variable	*Inveff*1	*Inveff*2	*Inveff*3	*Inveff*4
*GARCHOIL*	0.1090***	0.0833***	0.1163***	0.0631
	(3.9268)	(2.6082)	(3.1566)	(1.6419)
*Lev*	0.0027***	0.0033***	0.0029***	0.0038***
	(13.0672)	(14.0751)	(12.1508)	(14.5203)
*Q*	0.0005***	0.0005***	0.0005***	0.0005***
	(15.3970)	(12.7530)	(13.2461)	(11.3713)
*CF*	0.0010	0.0003	0.0019*	0.0010
	(1.1176)	(0.2554)	(1.7752)	(0.8031)
*ROA*	0.0225***	0.0149***	0.0218***	0.0173***
	(9.7262)	(5.5829)	(7.9599)	(5.7814)
*Colla*	0.0059***	0.0051***	0.0063***	0.0057***
	(24.4729)	(18.2882)	(22.3747)	(18.3614)
*Adm*	0.0000	-0.0008	-0.0001	-0.0005
	(0.0116)	(-1.5828)	(-0.1237)	(-0.8570)
*Size*	-0.0002***	-0.0003***	-0.0003***	-0.0004***
	(-5.6269)	(-6.3648)	(-7.5308)	(-8.2986)
*Age*	-0.0018***	-0.0012***	-0.0018***	-0.0012***
	(-44.2392)	(-24.7416)	(-35.6565)	(-21.3295)
*TOP*1	-0.0000	0.0000	-0.0000*	-0.0000
	(-1.0666)	(0.6342)	(-1.8224)	(-0.7727)
*Id*	0.0016**	0.0031***	0.0019**	0.0034***
	(2.3994)	(3.9410)	(2.3867)	(3.9499)
*Dual*	0.0005***	0.0005***	0.0005***	0.0005***
	(7.0670)	(6.2329)	(5.5348)	(4.8825)
*Dboard*	-0.0000	-0.0001**	-0.0000	-0.0001*
	(-0.9817)	(-2.3803)	(-0.2878)	(-1.7888)
*Sboard*	-0.0001**	-0.0001**	-0.0001*	-0.0001
	(-2.5693)	(-2.0206)	(-1.7169)	(-1.3328)
*GDP*	-0.0017	0.0011	-0.0011	0.0013
	(-1.3100)	(0.7472)	(-0.6151)	(0.6472)
_*cons*	0.0162***	0.0198***	0.0186***	0.0219***
	(19.1832)	(20.4349)	(19.1342)	(20.4554)
*Industry*/*Time*	Yes	Yes	Yes	Yes

### 5. Further analysis

#### (1) Life cycle

Firm’s internal cash flows and external financing sources change systematically with the passage of the firm’s life cycle. Therefore, this paper further explores whether the impact of oil price uncertainty on investment efficiency is affected by life cycle differentiation. The life cycle theory divides the growth and development trajectory of enterprises into three periods: growth, maturity and recession. Firms in the growth period have just entered the market with a single business, a small production scale, a lack of internal cash flow, a large degree of external financing constraints and tight sources of capital [44]. Conservative investment decisions are taken in the face of the risk of oil price uncertainty, which will result in underinvestment. Enterprises in the maturity stage occupy a stable market position, with abundant endogenous funds and smooth channels of exogenous financing. But the managers of the enterprise have the motive of overinvestment behaviors for their welfare [45]. In this stage, the impact of oil price uncertainty on investment efficiency will be strengthened by the management’s self-interest motive. Underinvestment occurs during the recessionary period when firms are trying to preserve the only remaining market share [46]. But if businesses continue to cut back on their investments and fail to seize the favorable investment prospects brought on by oil prices uncertainty, their profit margins will contract, increasing their risk of going bankrupt. When an enterprise is bankrupt, liquidation will harm the implicit welfare of management and the interests of all participants [45], so it is more urgent to shrewdly allocate the remaining funds to make investments that will give the enterprise a competitive edge and reduce its propensity to be merged, acquired, or delisted. Based on the above analyses, it can be inferred that the degree of inhibition of investment efficiency by oil price uncertainty is more significant in the growth and maturity periods than during the recession period.

Based on the characteristics of the life cycle of the firms, concerning Jian Ma and Shu Lin (2021) [46], the sample is divided into three groups: growth, maturity and recession periods. The first three columns of Table 6 show the moderating effect of the life cycle on oil price uncertainty and investment efficiency. The GARCHOIL coefficient is 0.083, which is significantly positive at the 5 percent level when in the recession period. The GARCHOIL coefficient is 0.130, which is significantly positive at the 1 percent level when in the maturity period. The GARCHOIL coefficient is 0.148, which is significantly positive at the 1 percent level when in the growth stage. According to the regression results, the impact of oil price uncertainty on investment efficiency is affected by life cycle differentiation and the inhibitory effect of oil price uncertainty on investment efficiency is weaker when in the recession period.

**Table 6:** Results based on life cycle, nature of property rights, and enterprise size.

	Panel A Life Cycle			Panel B Nature of Property Rights		Panel C Enterprise size	
	recession	maturity	growth	non-state	state	small	large
*GARCHOIL*	0.083**	0.130***	0.148***	0.117***	0.088**	0.122***	0.070
	(2.35)	(3.05)	(2.79)	(3.52)	(2.18)	(4.01)	(1.42)
_*cons*	0.016***	0.016***	0.017***	0.010***	0.018***	0.019***	0.020***
	(13.35)	(11.59)	(11.43)	(7.99)	(16.15)	(12.71)	(11.67)
*Conrtols*	Yes	Yes	Yes	Yes	Yes	Yes	Yes
*Industry*/*Time*	Yes	Yes	Yes	Yes	Yes	Yes	Yes
N	30273	26486	34257	57040	33976	68475	22541
F	114.733	60.322	82.806	144.202	82.847	182.864	57.194
R^2^	0.1400	0.0941	0.0873	0.0933	0.0998	0.0942	0.1105

#### (2) Nature of property rights

Due to China’s unique political system, there are two types of firms in the market economy: state-owned and non-state-owned. There are certain differences in the impact of oil price uncertainty and investment efficiency because of the two different property rights natures of the enterprises and the benefits they enjoy and the responsibilities they take. State-owned enterprises (SOEs) have a high ability to resist risks due to their highly profitable operation, close association with the government and other advantages [9]. Compared with non-state-owned enterprises, the impact of the risk of oil price uncertainty on state-owned enterprises is likely to be hedged, and SOEs have insufficient incentives to postpone investment. What’s more, the policy burden, social responsibility, and people’s supervision brought by the state-owned system [46] lead to a higher cost for SOEs to exploit oil price uncertainty for private gain. Therefore, this paper thinks: non-SOEs will inhibit the negative correlation of oil price uncertainty on investment efficiency.

To verify the differential impact of the nature of property rights, this paper divides the sample into state-owned enterprise groups (SOE = 1) and non-state-owned enterprise groups (SOE = 0) to conduct multiple regression tests, and the specific results are shown in Table 6. In the data of the state-owned enterprise sample, the coefficient of oil price uncertainty on investment efficiency is 0.088, which is significant at the 5 percent level. While the coefficient of non-state-owned enterprises is 0.117 and is significantly positive at the 1 percent level. It can be concluded that the inhibitory effect of oil price uncertainty on investment efficiency is affected by the nature of the enterprise’s property rights, and that non-state enterprises have more difficulties maintaining existing assets or positive NPV projects when facing the risk of oil price uncertainty and tend to downsize their investments even more, which makes their enterprises even less efficient.

#### (3) Enterprise size

Financing is a guarantee for the smooth operation of production and investment activities, but external financing is regulated by the size of the enterprise. Medium- and small-sized enterprises are significantly behind the curve in terms of technology, have relatively little capital, experience discrimination in the bank credit system, and have limited access to external financing alternatives [9]. The uncertainty of oil prices leads to a shortage of endogenous funds if the original level of investment continues to be maintained, the medium and small sized enterprises will face a crisis of bankruptcy. Therefore, the medium and small sized enterprises facing the risk of oil price uncertainty are more willing to reduce the scale of investment and lower investment efficiency. In addition, high oil price uncertainty may increase the default risk of enterprises. External investors in security considerations adopt the tight monetary policy, resulting in a reduction in the number of enterprises to obtain external funds [38]. The medium and small sized enterprises will increase their cash holding reserves due to future uncertainty considerations, which further exacerbates the inefficient investment in oil price uncertainty. As a result, it can be expected that the dampening effect of oil price uncertainty on investment efficiency is more significant in the medium and small sized enterprises relative to large enterprises.

In this paper, the natural logarithm of total assets is used to classify firm size. A dummy variable for size SIZE is created, with firms above the highest quartile classified as large-scale firms (SIZE = 1) and conversely as small- and medium-scale firms (SIZE = 0). The last two columns of Table 6 show the regression results. For small and medium-sized firms, the coefficient of oil price uncertainty is significantly positive. And every 1% increase in oil price uncertainty causes a 12.2% decrease in investment efficiency. In large-scale enterprises, the regression coefficient of oil price uncertainty is positive but not significant, which indicates that the size of the firm mitigates the inhibitory effect of oil price uncertainty on investment efficiency, which means that the negative effect of oil price uncertainty on investment efficiency in large-scale enterprises is significantly weaker than that of small and medium-sized enterprises.

### 6. Conclusions

This paper uses a model with quarterly panel data of China’s A-share listed companies from 2011 to 2020 to empirically analyze the relationship between oil price uncertainty and corporate investment efficiency.

The results of the study are as follows: Oil price uncertainty is negatively correlated with corporate investment efficiency in the full sample. The mechanism of oil price uncertainty on overinvestment and underinvestment is further examined in the paper. It is found that the "oil price uncertainty—cash holding—overinvestment—investment efficiency" transmission pathway is effective. In addition, the above-mentioned influence is also regulated by China’s special property right background, enterprise scale and life cycle, which is specifically manifested in the fact that this inhibition is more significant in non-state-owned enterprises, small and medium-sized enterprises, enterprises in growth and maturity stages.

Based on the above conclusions, from the governmental perspective, the government should promote reform of the energy structure, actively guide enterprises to use new energy sources and reduce their dependence on crude oil. At the same time, government should also increase the supervision of enterprises from the legal level, encourage enterprises to actively disclose information about their investment activities, reduce the degree of information asymmetry, and promote effective investment. From the perspective of enterprises, firstly, the management should strictly control the prior risk prediction of investment activities, and reasonably avoid the risks brought by the uncertainty of oil prices. Secondly, the investment decisions of enterprises must be vertically audited. Cannot only listen to the opinions of management or major shareholders to ensure the rationality of investment. Last but not least, the enterprise should improve the relevant regulatory mechanism, strengthen the exchange of information among stakeholders, reduce the management’s inefficient investment behavior, and reasonably cope with the risks and opportunities brought by global oil price uncertainty.

When researching the factors that influence investment efficiency, the majority of academics used to primarily concentrate on corporate governance mechanisms, information disclosure, corporate management, and the economic policy environment [12–17]. They hardly ever addressed the effect that market uncertainty on enterprise production factors has on investment efficiency. This study examines the effect factors of corporate investment efficiency from the perspective of global oil price uncertainty in an effort to further enhance the research framework on corporate investment efficiency.

This study delves into the relationship between oil price uncertainty and the investment efficiency of domestic enterprises. While it adds the moderating variable of executive compensation gap, it also reveals the facilitating effect of oil price uncertainty on investment efficiency and enriches the research framework of oil price uncertainty and investment efficiency.

The following deficiencies remain: the limitations in the selection of investment efficiency, the omission of the executive team’s own characteristics when studying the moderating effect of the executive pay gap, and the limitations of the decomposition of oil price fluctuations. These are due to the missing values of some data in the empirical test, the imprecise way of measuring the relevant variables, and the lack of relevant theoretical mechanisms. This issue is not well supported by theory or empirical data, and future research may focus on using a more persuasive indicator to depict the behavior of the global economy.
